# Beyond Efficiencies: Guiding Artificial Intelligence in the Planning and Design of Healthcare Facilities

**DOI:** 10.1177/08404704261418915

**Published:** 2026-02-06

**Authors:** Stephen Verderber, Ramsey Kin-Sun Leung

**Affiliations:** 1 Dalla Lana School of Public Health/Institute of Health Policy, Management and Evaluation, 7938 University of Toronto, Toronto, Ontario, Canada; 2 John H. Daniels Faculty of Architecture, Landscape and Design, 152805 University of Toronto, Toronto, Ontario, Canada; 3 Centre for Design + Health Innovation, 152805 University of Toronto, Toronto, Ontario, Canada

## Abstract

Artificial intelligence for health holds enormous transformative potential. It has already proven successful in enhancing patient outcomes, facilitating the duties of professional caregivers, and their organizations. Its potential applications in the field of healthcare architecture are similarly being explored. This article discusses the role and function of generative artificial intelligence with respect to the professionals who work in close collaboration with healthcare organizational clients and their direct-care constituencies in decision framing the planning, design, construction, and management of healthcare facilities. Opportunities and challenges associated with generative artificial intelligence in the facility procurement process are discussed, including the role of ethics and societal responsibility at this critical juncture.

## Introduction

### Architecture for Health

Healthcare organizations are being presented with generative Artificial Intelligence (AI)-based case studies in nearly every aspect of clinical and operational practice, and the design of healthcare facilities is no exception in this trajectory. Architectural and engineering teams working in the healthcare sector are experimenting with generative AI-enabled tools in the planning, forecasting, and evaluation of patient care physical environments. These assistive tools are arriving at a moment when healthcare systems face mounting pressures including ageing facilities, associated infrastructure, shifting models of care, workforce shortages, and rising expectations from patients and local communities.^1^ As the rate of generative AI-based technological change accelerates, health leaders are searching for the means and methods to use this new technology to anticipate their organizational needs.^2^ This includes the need to accurately shepherd the design, construction, and daily management of their facilities and related infrastructural systems to remain highly clinically relevant.

A growing challenge in healthcare facility planning and design is the widening dissonance between the relatively slow pace in the generation of building bricks-and-mortar facilities vs. increasingly accelerated developments occurring in AI-assisted clinical practice. Medical devices are evolving at an ever-faster pace than the building infrastructures designed to facilitate them, with the advent of generative AI-enabled systems threatening to further compound and widen this gap. Hospitals can no longer be assumed to retain long-term functional alignment relevance of their physical layouts, with healthcare organizations experiencing significant functional misalignments less than a decade following initial occupancy due to advances in both technology and care delivery best practices.^3,4^ Such uncertainties might be mitigated by effectively, creatively, utilizing generative AI tools in the iteration and analysis of multiple design options, patient flow variances, and departmental adjacencies with far greater efficiency than at present. These tools are nascent, however, and are still approached with some trepidation by many Architecture/Engineering (A/E) firms.

While some firms now use generative AI to quickly iterate alternate functional layouts, early results of this experimentation highlight inherent risks alongside multiple uncertain benefits. Currently unresolved issues surround questions of data security, transparency, interpretability, and trustworthiness that will likely require some degree of regulatory oversight.^5^ But even in this early adoption stage, generative AI influences how facility planners and designers are thinking about processes and priorities. Beyond questions surrounding its implementation, the advent of generative AI-enabled tools raises broad ethical questions surrounding health equity, public safety, societal responsibility and expectations, ecological determinants, and environmental responsibility.^6-8^

The aim of this discussion is to review a set of key emerging issues surrounding generative AI in the planning and design of healthcare facilities drawing on recent disciplinary literature across healthcare management, architecture, and AI ethics. Next, potential advantages, risks, and regulatory opportunities to govern the judicious, creative, cost-effective use of these tools are briefly outlined as applicable in everyday use contexts. While not yet a central actor in healthcare facility planning and design, the trajectory of its rapid adoption in all walks of life suggest that it will come to directly influence day to day patient care physical environments. As such, both healthcare architects and allied professionals and health leaders and allied professionals can be advantaged through literacy in this new technology to most effectively maintain control of this technology with clarity. This set of issues is meant to be viewed as a prism to discern effective strategies in everyday professional practice. Throughout, the focus remains on how generative AI intersects with the human, ethical, and relational dimensions of healthcare built environments. Taken analytically, the integration of generative AI into healthcare facility planning can be understood through three interrelated leadership responsibilities. First is *oversight*: leaders must ensure that AI-supported decisions remain transparent, interpretable, and aligned with clinical and organizational priorities. Second is *accountability*: while AI tools may inform planning decisions, responsibility for outcomes—ethical, financial, and clinical—remains with human decision-makers. Third is *stewardship over time*: because facilities outlast technologies, leaders must assess how AI-enabled choices shape adaptability, resilience, and long-term care delivery. These three dimensions provide a practical framework for interpreting both the opportunities and risks discussed below.

## Opportunities and Risks

**Figure 1.**
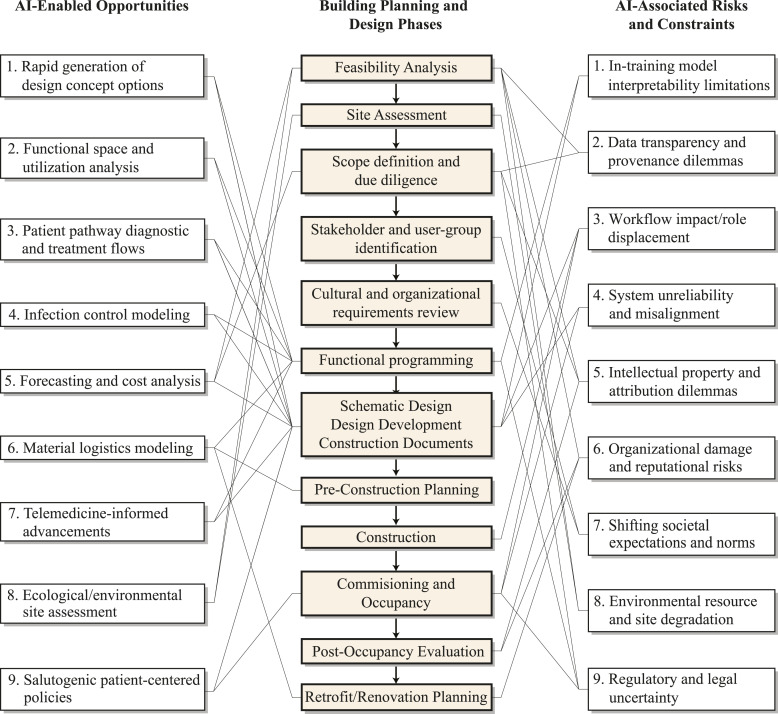
Potential intersections between AI-enabled tools and the healthcare facility planning and design process

Generative AI is transforming industry-wide expectations around how quickly healthcare facilities are planned, designed, built, and managed. This new technology is reshaping clinical practices, and patient care standards of excellence. For decades, many healthcare organizations have planned their buildings and associated infrastructural obsolescence rates on the assumption of a 50-year functional lifespan. This assumption is being eroded. With digital infrastructures accelerating so quickly, the functionality timeline period during which a given facility remains fully aligned with everyday clinical best practices is dramatically compressing. Shifting, accelerated best practice paradigms exert new pressures on both healthcare organizations and the A/E teams they contract with to work more collaboratively, continuously, from project start to finish and beyond. Figure 1 illustrates some basic interrelationships between the manifold of opportunities, as well as key risks, associated with generative AI-enabled tools as they transact and intersect with skill sets and proclivities involved in the work of healthcare-specialized facility planning and architectural design teams. Rather than functioning as a linear workflow diagram, Figure 1 is intended as a conceptual map of interdependencies as well as indeterminacies illustrating how opportunities and risks associated with generative AI recur and intersect across multiple phases of the facility’s planning and design. Generative AI tools promise accelerated performance expectations, including:

1. *Rapid Concept Generation*: Faster generation of early conceptual design options: AI systems can produce multiple test-fit alternatives, helping teams explore layouts that might otherwise take days or weeks to iterate, resulting in accelerated design creativity, and is listed in Figure 1, Left Column, as an Opportunity (OP): F1-LC: OP 1, 9.
2. *Refined Facility Functionality*: Examination of optimal staff, patient, and visitor patterns of use and movement within the unit, infection control optimization, and spatial adjacencies. Generative AI systems may alter the traditional space requirements at the scale of individual departments, such as materials management, telemedicine units, and inpatient care areas via salutogenic design features (F1-LC: OP 2, 9).
3. *Expedited Clinical Workflows*: Generative AI is being used to visualize diagnostic and treatment pathways, that is, embedded anticipatory cueing, impacting traditional adjacency design choices around a triangulation of circulation, infection control measures, modality-specific equipment placement, and caregiving team proximities, resulting in significant patient diagnostic and treatment improvements (F1-LC: OP 3-4, 9).
4. *Forecasting and Cost-Benefit Improvements*: These tools can analyze historical market data and current supply-chain conditions to produce more accurate early cost estimates and assess the fiscal planning implications of strategic project scope modifications (F1-LC: OP 5-7, 9).
5. *Site and Environmental Assessment Improvements*: Early-stage evaluation of topography, climate, zoning, and ecological determinants can be conducted more expediently, narrowing decision windows in the pre-design feasibility assessment phase (F1-LC: OP 8-9).

Generative AI adoption is now occurring broadly within the A/E sector, although still highly inconsistently and often largely exploratory. As Wardon notes, A/E firms, including Abramson Architects, are experimenting with how AI can streamline the generation of design options with regards to space utilization, operational flow, efficiency, and operational cost containment, yet these efforts are still in their infancy from an research and development perspective.^9^ Walch makes similar predictions for its adoption in broader architectural practice, with these tools projected to aid in the evaluation of a given site including its topography, zoning regulation analysis, climate data, and ecological determinants to help identify in minutes what once would have previously required weeks of laborious analysis and reporting time.^10^ By being virtually immediately able to analyze historical pricing data, current market conditions, and material cost-feasibility trends, more accurate estimates are available from earliest capital improvement project stages. And if a capital improvement project’s scope shifts (as so often happens) these tools can comprehensively forecast modified associated cost and schedule ramifications in real time.

Yet the usefulness of these tools depends entirely on the quality and provenance of the inputted data assumptions. A far broader debate (beyond the scope of this discussion) concerns the systemic human biases of large baseline training datasets, but bracketing this aside, healthcare specific and architecture specific datasets must be accurately inputted. Without this critical step, generated output risks being misleading, misdirecting, or structurally pre-biased with potentially high probability of compromising organizational workflow efficacy, health outcomes, ethical, and ecological/environmental outcomes. In healthcare facility planning and design, several risk factors therefore deserve intensive attention, including:

1. *Data Non-Interpretability*: Healthcare facility planners and designers may be confronted with the inability to definitively trace the reasoning behind a generative AI model’s recommendations, thereby polluting the interpretability quotient of the system’s output. This condition is often referred to as system untrustworthiness, unpredictability, and uncontrollability (F1-Right Column: Risks/Constraints 1-2).
2. *Data Vulnerabilities*: Cybersecurity controls may be fragile and thin, and a given generative AI model may be deeply flawed intrinsically, or tampered with by a third party. Either scenario may result in misalignment challenges insofar as outputs would not match the users’ intended inputted goals. Also, the threat of an organization’s defamation by a competitor is real, as well as the potential for extensive intellectual property issues (F1-RC: Risks/Constraints 2-6).
3. *Significant Job Loss and Displacement*: As with any major technological shift, jobs losses are inevitable, particularly for workers engaged in repetitive, routine tasks. While healthcare-specialist architects shouldn’t expect to be replaced *en masse* in the near future, generative AI adoption will reduce the demand for certain entry-level technical subfields within the architecture and environmental design professions.^11,12^ Nonetheless, in some sectors within the A/E industry significant job displacement is likely in the next decade (F1-RC: Risk/Constraints 2-4).
4. *Ineffective Regulatory Control Guardrails:* As guidelines governing AI applications in clinical and in facility planning contexts continue to evolve, the risk is great insufficient oversight by regulatory agencies will be the *modus operandi* at the local, regional, and federal levels. Harmful, misaligned, non-health promoting outcomes could result. This can result in environmental degradation ranging from facility-level (micro) issues to broader societal (macro) norms and expectations. Regulatory agencies with oversight of the design, construction, and management of healthcare facilities will need to enact guardrails (F1-RC: Risks/Constraints 7-9).
5. *System Sycophancy*: The threat is real a given generative AI model will give its users only what it thinks they want to hear. This can knowingly or unknowingly result in useless output. Effective cybersecurity safeguards are needed to protect against this occurrence. Any and all of the above listed threats and challenges are intrinsically related to this operative condition. If unchecked, these generative models may produce confident but incorrect outputs that appear authoritative (F1-RC: Risks/Constraints 6-9).

As mentioned, architectural practitioners in an A/E firm that rely on routine calculations or administrative repetition are vulnerable to elimination due to AI-induced automation. Repetitive yet critical tasks including structural engineering, material analysis and the writing of project specifications, the development of myriad building assembly details, and the management and coordination of a facility construction project could, in theory, be readily automated. Yet, generative AI is also capable of formulating a building’s entire material lifecycle from cradle-to-cradle as part of the growing trend toward circular restreaming and regenerative recycling-based global economies. For healthcare organizations, such strategies could be part of a wider obligation to support reconfigurable and resilient caregiving paradigms. Its manifold influences requires each healthcare organization to be equipped to engage with and balance potential efficiencies accruable vis-à-vis this technology against ethical and relational ramifications and unexpected outcomes relative to the planning, design, and construction of built environments for healthcare. Viewed through this governance lens, the opportunities and risks of generative AI are not merely technical variables, but indicators of where oversight, accountability, and long-term stewardship must be actively exercised by health leaders.

## Governance, Trust, and Ethical Use

Governance of these tools is central to ensuring the public’s trust in their appropriate adoption. The decisions made by a given organization during earliest planning stage of a healthcare capital improvement project can have far-reaching legal and regulatory ramifications later. But the current legal and regulatory landscape remains highly random. However, recent legislation in Europe such as the E.U.A.I. Act outline several foundational principles related to data transparency, data protection, and accountable human oversight, among other *guardrailing* measures. Comparable regulatory efforts in North America are yet to appear. The questions that matter most from a healthcare facility planning and design perspective (liability, negligence, intellectual property, and best practice standards of professional care) are currently being worked out *ad hoc* in real time often through litigation or within-industry sector specific random guidance. As legal precedents in due time are established, architects and healthcare organizations alike must rely on self-regulation and *ad hoc* avenues of recourse when adverse facility design outcomes occur due to faulty or opaque generative AI models.

These concerns, of course, extend across all aspects of society. As Nasarian et al. note,^13^ there is a difficulty in determining whether any specific AI model has been tampered with, potentially undermining trust and confidence. Even subtle misalignments in generative model training can lead to biased outputs, or exhibit behaviour difficult for decision-makers to accurately interpret. Further downstream, Bridgeman et al. note^14^ the challenge for educators at all levels is in large part curatorial: centred in reliable input-output protocols and interpretation of generative AI output. But this is precisely, long term, where the effective controls and uses of this technology must begin.

The danger is real that humans will require, in the future, new ways to reinforce and refresh our AI-induced eroded cognitive abilities. But organizations that rush to future-proof their teams risk premature, incorrect, non-strategic responses. Effective adoption depends not only on technical safeguards but also the cultivation of human judgment to situate AI outputs within the lived realities of clinical care. The human skills most needed—creative thinking, wisdom accrued through prior experience, the capacity to learn new things, and flexible thinking styles—are now at risk. Two questions: “What thinking and creative reasoning abilities will human stakeholders in the healthcare facility procurement process need to thrive?” And second, “Will the widespread use of generative AI actually enhance those human abilities?” For example, if unverifiable tools and output were to inform decisions about a hospital ICUs patient flow, staffing assumptions, or safety-related design features, the consequences could be harmful if not catastrophic. Vigilance, reliable verification processes, and continuous organizational competency assessment metrics will be critical. As Bensinger has reported,^15^ the concerns of an organization’s reputational damage stemming from erroneous output are not hypothetical because misleading or false outputs related to a facility’s performance could erode public trust in that healthcare institution.

The ecological ramifications are also worth noting. The arrival of large-scale generative AI data centres is resulting in long-familiar environmental health inequities, particularly in communities lacking political leverage, or those already living with adverse environmental health outcomes, such as heightened cancer rates. Recent investigative reporting by a not-for-profit environmental agency, STAND.earth, has traced how the rapid expansion of these centres in rural communities is consuming high levels of local electrical energy and a high level of water consumption, further exacerbating existing public and personal health inequities.^16^

## Conclusion

Healthcare organizations, their commissioned architects, and allied design and engineering professionals who engage with this new technology face a shared ethical responsibility.^17^ Encouragingly, the American College of Healthcare Architects has begun to outline what responsible AI-assisted A/E practice might look like, including the need for extensive-intensive human oversight and control, transparent model documentation, verification of data outputs, and structured ethical review protocols.^18^ This appraisal process is occurring in architectural education as well.^19,20^ But as for healthcare organizations and their contracted A/E teams, AI literacy and governance frameworks are necessary to enable rational assessment of whether and to what extent this technology is suitable for a specific building’s design. Above all, will its use support rather than undermine the organization’s commitment to its patients, staff, the local community, and beyond? The contribution of this review lies in framing generative AI in healthcare facility planning not simply as a technical innovation, but as a socio-technical intervention whose consequences depend on governance, professional judgment, and ethical stewardship rather than algorithmic capability alone. This discussion is limited by its reliance on rapidly evolving literature and early-stage applications, and future empirical work will be required to assess how these dynamics unfold in practice. The future extends beyond basic questions of risk, efficiencies, or mere data output optimization. It also invites a reconsideration of how healthcare environments evolve over time, and how design teams can guide AI to support forms of care that remain highly adaptable, contextually specific, and environmentally responsible. What types of care will our built environments for health make possible and will more therapeutically support through the use of this new technology?^21,22^

For health leaders, the practical implication of this review is the need to establish governance structures that ensure human oversight, contextual validation, and ethical accountability when deploying generative AI tools in facility planning, cost forecasting, and site analysis workflows. In this context, salutogenic architectural design excellence for health expresses broadly ethical and relational concerns beyond merely the tectonic and functional.^23,24^ As healthcare architectural specialists and their clients adopt much greater use of generative AI it must be treated as a *non-neutral* assistive instrument. It impacts in highly diverse ways, involving patients, providers, infrastructures, institutions, communities, and their socio-cultural and ecological interrelationships. Inspirationally built and humanely guided by humans, this new technology will aid in reimagining healthcare built environments more intrinsically attuned to everyday realities and their broader long-term contexts. This will require comprehensive vision—and the reconsideration of what we mean by *care*.
